# The Yeast *PNC1* Longevity Gene Is Up-Regulated by mRNA Mistranslation

**DOI:** 10.1371/journal.pone.0005212

**Published:** 2009-04-17

**Authors:** Raquel M. Silva, Iven C. N. Duarte, João A. Paredes, Tatiana Lima-Costa, Michel Perrot, Hélian Boucherie, Brian J. Goodfellow, Ana C. Gomes, Denisa D. Mateus, Gabriela R. Moura, Manuel A. S. Santos

**Affiliations:** 1 Department of Biology and CESAM, University of Aveiro, Aveiro, Portugal; 2 CICECO & Department of Chemistry, University of Aveiro, Aveiro, Portugal; 3 Institut de Biochimie et Génétique Cellulaires, CNRS, Bordeaux, France; University of Minnesota, United States of America

## Abstract

Translation fidelity is critical for protein synthesis and to ensure correct cell functioning. Mutations in the protein synthesis machinery or environmental factors that increase synthesis of mistranslated proteins result in cell death and degeneration and are associated with neurodegenerative diseases, cancer and with an increasing number of mitochondrial disorders. Remarkably, mRNA mistranslation plays critical roles in the evolution of the genetic code, can be beneficial under stress conditions in yeast and in *Escherichia coli* and is an important source of peptides for MHC class I complex in dendritic cells. Despite this, its biology has been overlooked over the years due to technical difficulties in its detection and quantification. In order to shed new light on the biological relevance of mistranslation we have generated codon misreading in *Saccharomyces cerevisiae* using drugs and tRNA engineering methodologies. Surprisingly, such mistranslation up-regulated the longevity gene *PNC1*. Similar results were also obtained in cells grown in the presence of amino acid analogues that promote protein misfolding. The overall data showed that *PNC1* is a biomarker of mRNA mistranslation and protein misfolding and that *PNC1-GFP* fusions can be used to monitor these two important biological phenomena *in vivo* in an easy manner, thus opening new avenues to understand their biological relevance.

## Introduction

Translation fidelity assures the production of stable and functional proteomes, but it is not an error free biological process as missense, frameshifting and nonsense errors can all interfere with mRNA translation accuracy [1]–[3]. Systematic quantification of these errors has not yet been carried out, but in *E. coli*, under normal physiological conditions, average missense error is of the order of 10^−3^ to 10^−4^ [1], while frameshifting and nonsense errors may be one order of magnitude higher. Missense errors in *E. coli* vary widely between codons and an important source of such variation is tRNA competition at near cognate codons [3]. Indeed, one recent study showed that *E. coli* tRNA_UUU_^Lys^ misreads near-cognate codons, positioned in the active site of firefly luciferase (Lys529; AAA and AAG), at frequencies of 3.6×10^−3^ to 2.0×10^−2^, which represents 18 fold difference between codons. A complementary study showed that misreading of leucine codons as histidine at position 45 of the α-subunit of the *Vibrio harveyi* luciferase varied from 2.0×10^−6^ to 1.5×10^−8^, which represents 133 fold variation among synonymous codons [4]. Moreover, codon context and environmental stress [5], [6] also influence decoding accuracy, creating a layer of complexity that complicates qualitative and quantitative analysis of mRNA mistranslation.

In eukaryotes, mistranslation also lacks systematic characterization, but *in vivo* in yeast, codon specific missense errors are of the order of 10^−5^ and *in vitro* in reticulocyte lysates are of the order of 10^−4^ [7]. Remarkably, studies carried out in HeLa and dendritic cells showed that 30% of newly synthesized proteins are aberrant and are rapidly targeted for degradation through the ubiquitin-proteasome pathway. Such defective ribosomal products (DRiPs) are a major source of presentation peptides for the MHC class I system and it is likely that mistranslation plays a critical role in surveillance of cell identity by the immune system [8], [9].

Other positive effects of mistranslation have been unravelled in studies on the evolution of genetic code alterations. In this case, codon misreading by mutant or wild type tRNAs works as a trigger for codon reassignment [10]–[13]. Such codon ambiguity also played a role during genetic code expansion from 20 to 22 amino acids and it is likely that it existed during the early stages of the development of the genetic code [14]. Finally, stop codon misreading generates morphological diversity in *S. cerevisiae* and CUG misreading generates extensive phenotypic variation in the human pathogen *Candida albicans* [15], [16].

The biological relevance of mistranslation is further highlighted during disease development and cell degeneration. For example, mRNA misreading in mitochondria is associated with severe myopathies [17]–[19], hypertension and dyslipidemia [20], while cytoplasmic misreading induces cellular degeneration and apoptosis in mammalian cells [21] and neurodegeneration in a mouse model [22]. It also triggers cell cycle defects and viability loss in *Schizosaccharomyces pombe* [23].

In order to shed new light into the biology of mRNA mistranslation, we have engineered constitutive codon misreading in *S. cerevisiae*, using a mutant tRNA that misreads leucine CUG codons as serine at 2.4%, which represents 240 fold increase in mistranslation relative to the typical error of 0,0001 [24]. Here, we show that such codon mistranslation in *S. cerevisiae* increased expression of the Pnc1 protein (Pnc1p) encoded by the *PNC1* gene. This gene plays an important role in aging because a yeast strain with 5 copies of *PNC1* lived 70% longer than the wild type strain [25]. This effect of Pnc1p is related to its enzymatic activity. Pnc1 synthesizes nicotinic acid from nicotinamide [26], which inhibits the NAD^+^-dependent histone deacetylase Sir2p [27] required for lifespan extension [27]–[30]. Lifespan extension occurs in response to calorie restriction, from yeasts to mammals, by mechanisms that are not yet fully understood [31]. In yeast, Sir2p is involved in chromatin silencing at telomeres, at ribosomal DNA (rDNA) and at mating type loci, and deletion of the *SIR2* gene promotes aging by increasing recombination at the rDNA locus [32]–[33]. However, recent data indicates that there are also Sir2p-independent pathways of lifespan extension [31], [34] and that Sir2p has a pro-aging role in yeast [35], implying that longevity may involve more complex mechanisms than those already known.

Pnc1p is induced in response to several environmental stressors, namely calorie restriction, heat shock or osmotic stress [25]. Here, we show that mRNA mistranslation in yeast, which is an intracellular stress, also induced expression and increased Pnc1p activity, which in turn activated Sir2p. We also show that Pnc1p could be induced by mistranslation inducing drugs and amino acid analogues, namely geneticin and canavanine, respectively, making it a potential biomarker of both mistranslation and protein misfolding.

## Results

### Pnc1 expression is induced by CUG mistranslation

In a previous study, we induced targeted and constitutive mRNA mistranslation in *S. cerevisiae*, using a tRNA_CAG_^Ser^ from the human pathogen *Candida albicans*, which decodes leucine CUG codons as serine. These recombinant yeast cells misincorporated 1.4–2.3% of serine randomly at the 30,994 CUG codons, which are distributed over 88.8% of its genes at an average frequency of 5.3 codons per gene, and had a global destabilizing impact on the proteome [15], [24].

**Figure 1:**
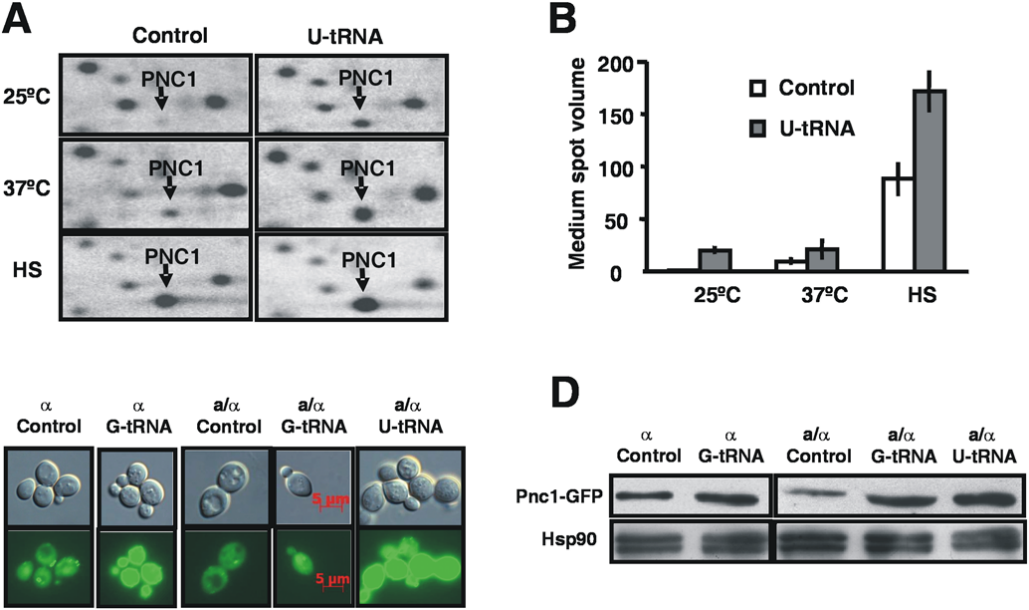
Pnc1 expression in CUG mistranslating cells. A) Details of a 2D-Map showing overexpression of the protein Pnc1. Proteins were labeled with [^35^S]-methionine and separated by 2D-PAGE. The portions of gels shown correspond to *S. cerevisiae* control cells or *S. cerevisiae* cells expressing the *C. albicans* U_33_ tRNA_CAG_^Ser^ (columns), grown at 25°C, 37°C, or under heat shock (lines). B) Quantification of Pnc1 expression by 2D-PAGE. *S. cerevisiae* control cells or *S. cerevisiae* cells expressing the *C. albicans* U_33_ tRNA_CAG_^Ser^ were grown at 25°C, 37°C, or under heat shock (HS), and mean spot volumes were normalized to the control value. Pnc1p was induced by all stress conditions and in both strains, but the fold increase was higher in CUG mistranslating cells. Results are expressed as mean±s.d. of three independent biological replicates. C) Pnc1 expression was induced *in vivo* in cells expressing the *C. albicans* tRNA_CAG_^Ser^. Epifluorescence microscopy showed enhanced fluorescence of a *PNC1-GFP* fusion protein in CUG mistranslating cells. D) Western blot using an anti-GFP antibody indicated increased expression from the *PNC1-GFP* fusion protein in mistranslating cells. The Hsp90 was used as a loading control. Signal quantitation is shown in Figure S2.

Quantitative proteomics using radiolabelled ^35^S-Methionine and 2D-PAGE protein fractionation indicated that Pnc1p was 30-fold up-regulated in response to CUG mistranslation, which was the highest level of protein up-regulation detected (Fig 1A,B). Pnc1p expression was further increased in mistranslating cells exposed to heat shock or grown at the sub-optimal temperature of 37°C (Fig 1A,B). However, microarray analysis indicated that Pnc1 expression was 2-fold increased in mistranslating cells only, suggesting that it is regulated post-transcriptionally (Figure S1; Data in ArrayExpress http://www.ebi.ac.uk/arrayexpress/ with the accession number E-TABM-196) [24]. In order to confirm *in vivo* the proteomics and transcriptomics data, we expressed GFP under the control of the *PNC1* promoter by engineering a PNC1-GFP fusion protein. Epifluorescence microscopy analysis of the recombinant yeast cells expressing the fluorescent reporter showed enhanced fluorescence of CUG mistranslating cells relative to controls (Fig 1C). In other words, the *PNC1* promoter was induced and the expression of the PNC1-GFP fusion protein was up-regulated. The Pnc1p overexpression was further confirmed by Western blot analysis of the fusion protein using an anti-GFP antibody (Fig 1D and Figure S2).

**Figure 2:**
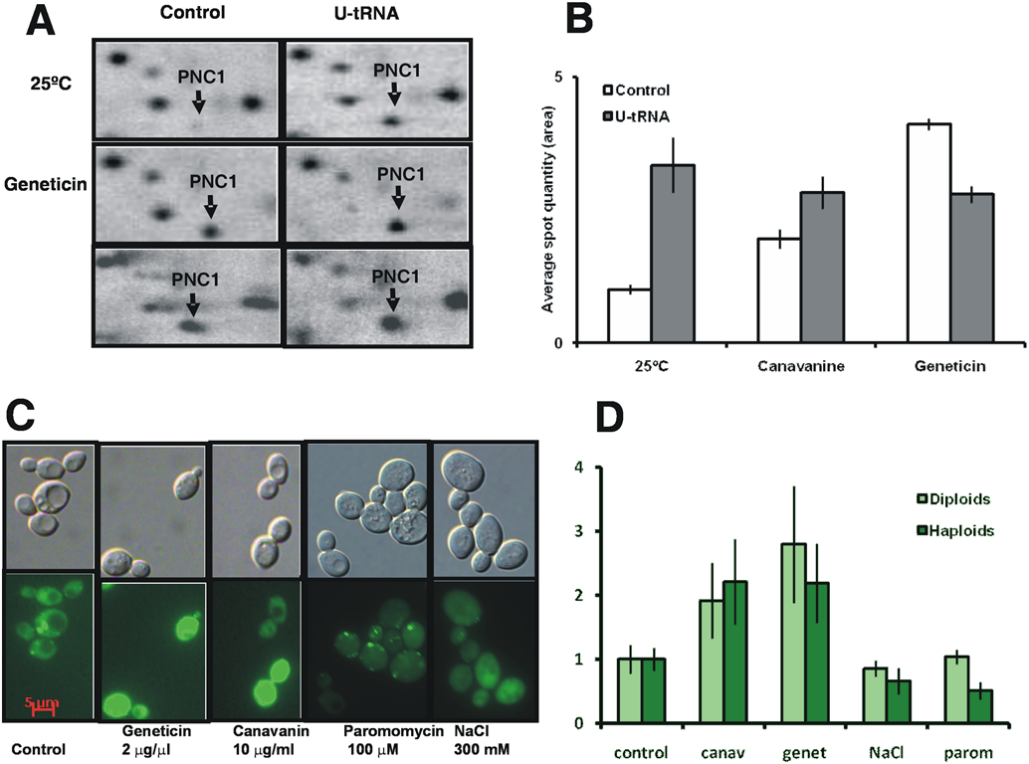
Pnc1 expression in response to drugs that induce mistranslation. A) Details of the 2D-Map showing the expression of the protein Pnc1, corresponding to *S. cerevisiae* control cells or *S. cerevisiae* cells expressing the *C. albicans* U_33_ tRNA_CAG_^Ser^ (columns), grown in the presence of 2 µg/µl geneticin and 10 µg/ml canavanine (lines) for 3 hours. Pnc1 was induced by all stress conditions and in both strains, but the fold increase was higher in CUG mistranslating cells. B) Pnc1p expression as determined by 2D-PAGE, showing that cells grown in the presence of the mistranslation-inducing drugs geneticin (2 µg/µl) and canavanine (10 µg/ml) also overexpressed Pnc1p. Results are expressed as mean±s.d. of three independent biological replicates. C) Pnc1 expression was increased *in vivo* in diploid cells in response to geneticin (2 µg/µl), canavanine (10 µg/ml), paromomycin (100 µM), and NaCl (300 mM). Epifluorescence microscopy showed enhanced fluorescence from the *PNC1-GFP* fusion protein (C, D). D) Quantification of PNC1-GFP induction in haploid and diploid cells. Results represent fluorescence intensity per cell, expressed as densitometric mean±s.d.

In order to determine whether Pnc1p overexpression was specific to CUG mistranslation or occurred in response to general mRNA mistranslation, cells were grown in presence of geneticin (2 µg/µl) and paromomycin (100 µM), and also in presence of the arginine analogue canavanine (10 µg/ml) whose incorporation into proteins results in misfolding [36]. Proteome characterization of these cells showed that geneticin and canavanine up-regulated Pnc1p expression, both in control and in cells mistranslating CUG codons (Fig 2A,B). Cells carrying the PNC1-GFP fusion protein submitted to the above drug treatments also displayed increased fluorescence indicating that Pnc1p expression was up-regulated (Fig 2C,D). Interestingly, paromomycin induced the formation of discrete fluorescent foci and there was no increase in total fluorescence, suggesting that Pnc1p was relocalized, which is consistent with previous studies that showed Pnc1p relocalization to peroxisomes under stress [25].

### CUG mistranslation increases the activity of Pnc1p and Sir2p

**Figure 3:**
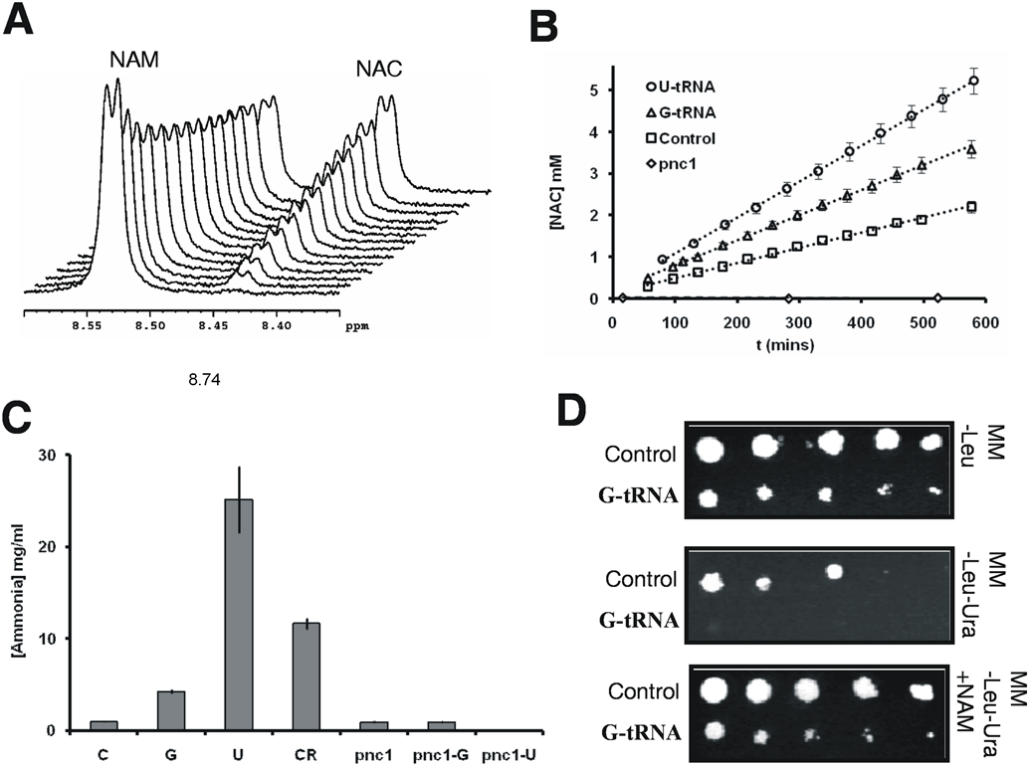
Pnc1 activity in CUG mistranslating cells. A) The conversion of NAM to NAC in control cell extracts spiked with NAM over a 600 min time period. The region of the ^1^H spectra containing the H6 protons of NAM (8.50 ppm) and of NAC (8.40 ppm) shows the conversion of NAM to NAC via a decrease in the area of the resonance from the H6 proton of NAM and a concurrent increase in the area of the resonance at 8.40 ppm from the H6 proton of NAC. B) Pnc1p activity was higher in response to CUG mistranslation as shown by a faster NAM conversion to NAC. NAC production was quantified by calibration using the area of the H6 proton in a 25 mM solution of NAM acquired under the same conditions as the spiked cell extracts. The peak areas obtained were also normalized to the sample with the lowest total protein content to allow for non-specific NAM/NAC binding. The rate of NAC production follows the order U-tRNA>G-tRNA>control. Spiked extracts from Δ*pnc1* cells showed no production of NAC over the time period of the experiments. C) Pnc1p activity was higher in CUG mistranslating cells, as shown by the increased release of ammonia. Values represent the difference in the quantity of ammonia produced in the presence or absence of nicotinamide. CR (calorie restriction) is included as a positive control. D) Silencing assays indicated that Sir2p activity was induced in CUG mistranslating cells. Silencing of the reporter gene *URA3* at the telomeres was shown by the inability of mistranslating cells to grow in minimal medium lacking uracil (middle panel), which was restored in the presence of nicotinamide that inhibits Sir2p (lower panel).

Pnc1p converts nicotinamide (NAM) to nicotinic acid (NAC), releasing ammonia during the reaction [26]. To elucidate whether Pnc1p up-regulation resulted in increased Pnc1p activity in CUG mistranslating cells, we quantified NAM and NAC in whole-cell extracts using NMR spectroscopy (Fig 3A,B). Comparison of the NMR spectra of NAM and NAC standard solutions with those of whole-cell extracts, acquired using a CPMG sequence to suppress broad lines, indicated that NAM and NAC levels were almost undetectable in CUG mistranslating cells (data not shown). Therefore in order to detect the presence of, and quantify Pnc1p activity, whole cell extracts were spiked with a standard NAM solution giving a final concentration of 25 mM. NMR spectra were then recorded every 5 minutes over a 600 minute time period. Figure 3A shows the region of the ^1^H spectra containing the H6 protons from spiked NAM (8.50 ppm) and from NAC (8.40 ppm) where NAM is being converted to NAC over time. The same procedure was carried out for extracts prepared from Δ*pnc1* cells where no increase in NAC levels (Fig 3B) over time were observed, confirming that Pnc1p is responsible for NAC production. The initial nonlinear decrease in NAM (Fig 3A) was seen in control cells and in Δ*pnc1* cells indicating that some non-specific binding of free NAM is occurring. To quantify NAC production a 25 mM solution of NAM was acquired using exactly the same conditions as for the spiked cell extracts and the area of the peak from the H6 proton used for calibration. The peak areas obtained were also normalized to the sample with the lowest total protein content to allow for non-specific NAM/NAC binding. A plot of free [NAC] versus time (Fig 3B) shows that the [NAC] in mistranslating cells is increasing at a faster rate than in controls. Assuming that NAC production is solely due to Pnc1p, we can conclude that Pnc1p is active in CUG mistranslating cells and its activity is higher in cells expressing the U_33_-tRNA_CAG_^Ser^, consistent with the increased expression of Pnc1p described above. Finally, nicotinamidase activity of Pnc1p was also assayed as described by Ghislain and colleagues and Anderson and colleagues [25], [26]. Ammonia release was higher in CUG mistranslating cells, whereas control and Δ*pnc1* strains showed low Pnc1p activity levels (Fig 3C).

Since Pnc1p regulates positively Sir2p in yeast [27], we wondered whether increased Pnc1p activity would result in increased Sir2p activity in CUG mistranslating cells. To test this hypothesis, we carried out gene expression silencing assays using the *URA3* reporter gene integrated at the telomeres [37]. Silencing was stronger in these cells since they were unable to grow in minimal medium lacking uracil, whereas the control cells could grow. This confirmed that Sir2p activity was also increased in CUG mistranslating cells (Fig 3D). However, when the medium was supplemented with NAM, Sir2p activity was inhibited and the silencing of the *URA3* gene was lost, allowing growth of both cell lines in minimal medium lacking uracil. This was consistent with the hypothesis that the induction of Pnc1p activity in CUG mistranslating cells increased Sir2p activity, which was reflected in enhanced telomere silencing.

## Discussion

This study showed an unexpected correlation between mRNA mistranslation/protein misfolding and up-regulation of the longevity gene *PNC1*. Since this gene is overexpressed under a variety of stress conditions, namely salt, sorbitol, nutrient restriction and heat stress [25], which are protein misfolding agents, and since the end points of mistranslation are protein misfolding, degradation or aggregation, it is likely that *PNC1* does not respond to mistranslation directly, but rather to increased protein degradation or to accumulation of aberrant proteins in the cytoplasm, ER or in organelles. In any case, *PNC1-GFP* fusions can be used to monitor mistranslation *in vivo* in absence of environmental stress. This is of biological relevance because mistranslation and protein misfolding *in vivo* are difficult to detect and quantify. Indeed, in our experience, detection of codon-specific mistranslation below 1% is technically difficult even with the most advanced mass-spectrometry methodologies. Conversely, the present study showed that 1.4% [24] of serine misincorporation generated strong fluorescence of the *PNC1-GFP* reporter system. Similar *PNC1-GFP* fluorescence results were obtained with 2 µg/µl of the misreading antibiotic geneticin and with 10 µg/ml of canavanine. Interestingly, the widely used mistranslation drug paromomycin resulted in *PNC1-GFP* fluorescence relocalization to discrete foci, indicating that *PNC1-GFP* may aggregate when mistranslated or that the stress induced by mistranslation may somehow result in PNC1-GFP relocalization to the peroxisome, as has already been observed in presence of other stress agents [25].

### The activation of Pnc1p and Sir2p by mistranslation

Our results showed that up-regulation of Pnc1p expression (Figs 1 and 2) resulted in increased Pnc1p and Sir2p activity (Fig 3) in mistranslating cells. In *S. pombe*, mRNA mistranslation resulted in abnormal cell division, aneuploidy and decreased cell viability [23] and these aneuploid cells had cell cycle defects [23], [38]. Considering that our previous studies, using flow cytometry analysis and DNA-microarrays, showed genome destabilization and cell ploidy alterations by mistranslation [24], we hypothesize that Pnc1p is necessary to maintain genome stability, through activation of the histone deacetylase Sir2p. Since the amount of Sir2p in the cell is limiting and critical to silencing and there is competition for this protein between silent regions of the genome [39], [40], we propose a model in which the redistribution of Sir2p to telomeres in mistranslating cells (to maintain genome stability) decreases its availability for silencing rDNA and mating-type loci, which can result in decreased replicative lifespan (unpublished data) and reduced mating efficiency of CUG mistranslating cells [24].

Pnc1p overexpression might also increase activity of additional histone deacetylases, besides Sir2p. In mammalian cells, Nampt, which is the functional homologue of Pnc1p [41]–[42], regulates the levels of mitochondrial NAD^+^, the activity of mitochondrial sirtuins and promotes cell survival under genotoxic stress [42]. Similarly, Pnc1p induction in mistranslating cells could prevent cell death and could maintain mitochondrial function by clearing nicotinamide inhibition and promoting NAD recycling and, thus, modulate the activity of other NAD^+^-dependent histone deacetylases. Since mRNA mistranslation increases under stress, during tumour development and in aging cells - conditions that are also characterized by genome instability [35], [43]–[44] - elucidating the role of Pnc1p and Sir2p activation in mistranslating unstable cells should be further investigated.

### Conclusions

We have shown that mRNA mistranslation induces the expression and increases activity of Pnc1p, a longevity gene that mediates lifespan extension in yeast. The results suggest that Pnc1p can be used as a molecular marker to detect mistranslation. This opens the door to monitor general and/or codon specific mistranslation *in vivo* under non-stress conditions using luminescent or fluorescent reporter proteins under the control of the *PNC1* promoter. Such systems could be used to monitor mistranslation in tumours, aging cells, neurodegenerative diseases, and mistranslation induced by antibiotics and other drugs that target the translational machinery. This is of biological and biomedical relevance because there is no simple methodology to monitor general mistranslation *in vivo* in both prokaryotic and eukaryotic cells.

## Methods

### Strains and growth conditions

*S. cerevisiae* strains used in this study were based on BY4742 (MATα; his3Δ1; leu2Δ0; lys2Δ0; ura3Δ0) and BY4743 (MATa/α; his3Δ1/his3Δ1; leu2Δ0/leu2Δ0; met15Δ0/MET15; LYS2/lys2Δ0; ura3Δ0/ura3Δ0) backgrounds and were acquired from EUROSCARF. Proteome characterization was performed in *S. cerevisiae* CEN.PK2 strain. Silencing assays were carried out using *S. cerevisiae* strain UCC3505 (kindly provided by Daniel Gottschling). Strains were transformed with the plasmids pRS315 (Control), pUKC715 (G_33_ tRNA_CAG_^Ser^) and pUKC 716 (U_33_ tRNA_CAG_^Ser^), described elsewhere [45]. Cells were grown in YEPD (1% yeast extract, 2% peptone, 2% glucose or 0.5% in calorie restriction experiments) or MM-leu (0.67% yeast nitrogen base, 2% glucose or 3% galactose, 0.2% drop-out mix containing all amino acids except leucine).

### Proteome Analysis

For quantification purposes, proteins were radio labeled *in vivo* with [^35^S]-methionine prior to 2D-PAGE analysis. 2D-electrophoresis and 2D-protein map analyses was carried out as described by Boucherie and colleagues [24], [46]–[48].

### Construction of the PNC1-GFP fusion protein

GFP was amplified from the plasmid pKT128 by PCR. Cells were transformed with 45 µl of the PCR reaction, using the lithium acetate method [49], and integrations on the *PNC1* locus were confirmed by PCR. Fluorescence was monitored by epifluorescence microscopy and the positive clones were re-transformed with plasmids as described above. Transformants were grown to OD = 0.5, scanned for GFP fluorescence and photographed under an Axio Imager.Z1 microscope (Zeiss).

### Western Blot Analysis

Proteins were detected using an anti-GFP antibody (Santa Cruz) and an anti-Hsp90 antibody, according to standard techniques.

### NMR

Exponentially-growing cells were harvested by centrifugation, the pellet was washed three times with mQ water and frozen at −80°C. Whole-cell extracts were prepared in lysis buffer (PBS, pH 7.0), in the presence of glass beads. Beads were washed with D_2_O and the supernatant collected. Lysates were cleared by centrifugation at 13000 rpm, for 10 minutes at 4°C. The extract was collected into a new tube and frozen at −80°C. The total protein concentration in the extract was determined using the Micro BCA Protein Assay Kit (Pierce). For NMR studies, samples were centrifuged at 13000 rpm, for 10 minutes and 600 µl were transferred into a 5 mm NMR tube. Spectra were acquired on a Bruker DRX500 spectrometer at 300K using the Bruker cpmgpr pulse sequence. A 16 ppm sweep width was used with a 3 s recycle delay and 128 scans were coadded.

### Nicotinamidase activity assay

Pnc1p activity was determined as described [25]–[26]. Briefly, whole-cell extracts were prepared from mid-exponential phase cultures by disrupting cells with glass beads on a MiniBeadBeater (Biospec Products), in homogenization buffer (10 mM Tris pH 7.5, 150 mM NaCl) supplemented with protease inhibitors (2 mM PMSF and EDTA-free protease inhibitor cocktail tablets from Roche). The total protein concentration of each sample was determined using the Micro BCA Protein Assay Kit (Pierce). 160 µg of protein (10–50 µl whole-cell extracts) were incubated with either 0 or 8 mM nicotinamide at 30°C, in a final volume of 400 µl of 10 mM Tris-HCl pH 7.5, 150 mM NaCl and 1 mM MgCl_2_, for 60 to 120 minutes. Pnc1p activity was calculated by measuring the ammonia concentration with an ammonia assay kit (Megazyme), according to the manufacturer's instructions.

### Silencing Assays

Silencing was performed as described [27], [37], [50]. Cells were grown to mid-exponential phase, serially diluted and plated in MM-Leu, MM-Leu-Ura and MM-Leu-Ura supplemented with 5 mM nicotinamide.

## Supporting Information

Figure S1 Comparison between transcriptomics and proteomics data on PNC1 expression in CUG mistranslating cells. DNA-microarray analysis showed that the PNC1 gene is induced 2-fold (data deposited in ArrayExpress http://www.ebi.ac.uk/arrayexpress/), whereas 2D-PAGE detected 30-fold increase in Pnc1p expression, suggesting that there is translational control of gene expression in CUG mistranslating cells. Microarray analyses were performed with 6 independent cultures for each strain and hybridized against the reference in dye-swap (three control strain cultures labelled Cy5 and three labelled Cy3), in a total of 6 microarrays for each mutant strain, as described previously (Silva et al, 2007). Data analysis was performed using GeneSpring (Silicon Genetics) and SAM (Significance Analysis for Microarrays). Comparison of GeneSpring data (P<0.05 by Student's t-test, fold change>1.6) and SAM analysis (D = 2.15; false discovery rate = 0.001) resulted in a common set of 170 significant genes, from which 81 were selected based on the average fold change. Proteome analyses were carried out for 3 independent biological replicates and the mean spot volumes were calculated after normalization to the total spot volume of the gel. Protein P-value was calculated using Student's t-test.
(8.43 MB TIF)

Figure S2 Quantification of the PNC-GFP fusion protein by densitometry. A) Fluorescence intensity was determined in the microscopy images using the AxioVision software from Zeiss. The area of each cell was delimited and the pixel intensity calculated. Values were normalized to the control and represent mean density±standard deviation. B) Western blot quantification was performed in the QuantityOne software from BioRad. Bands were delimited by boxes with similar area, and the densities calculated. After background subtraction, values of the GFP bands were normalized to the values of the HSP bands. Results are expressed as mean density±standard deviation.
(1.46 MB TIF)
