# Supplementary figures and images for: The Yeast PNC1 Longevity Gene Is Up-Regulated by mRNA Mistranslation
Source: PLoS One. 2009 Apr 17;4(4):e5212. doi: 10.1371/journal.pone.0005212 (PMC2667667; doi:10.1371/journal.pone.0005212)

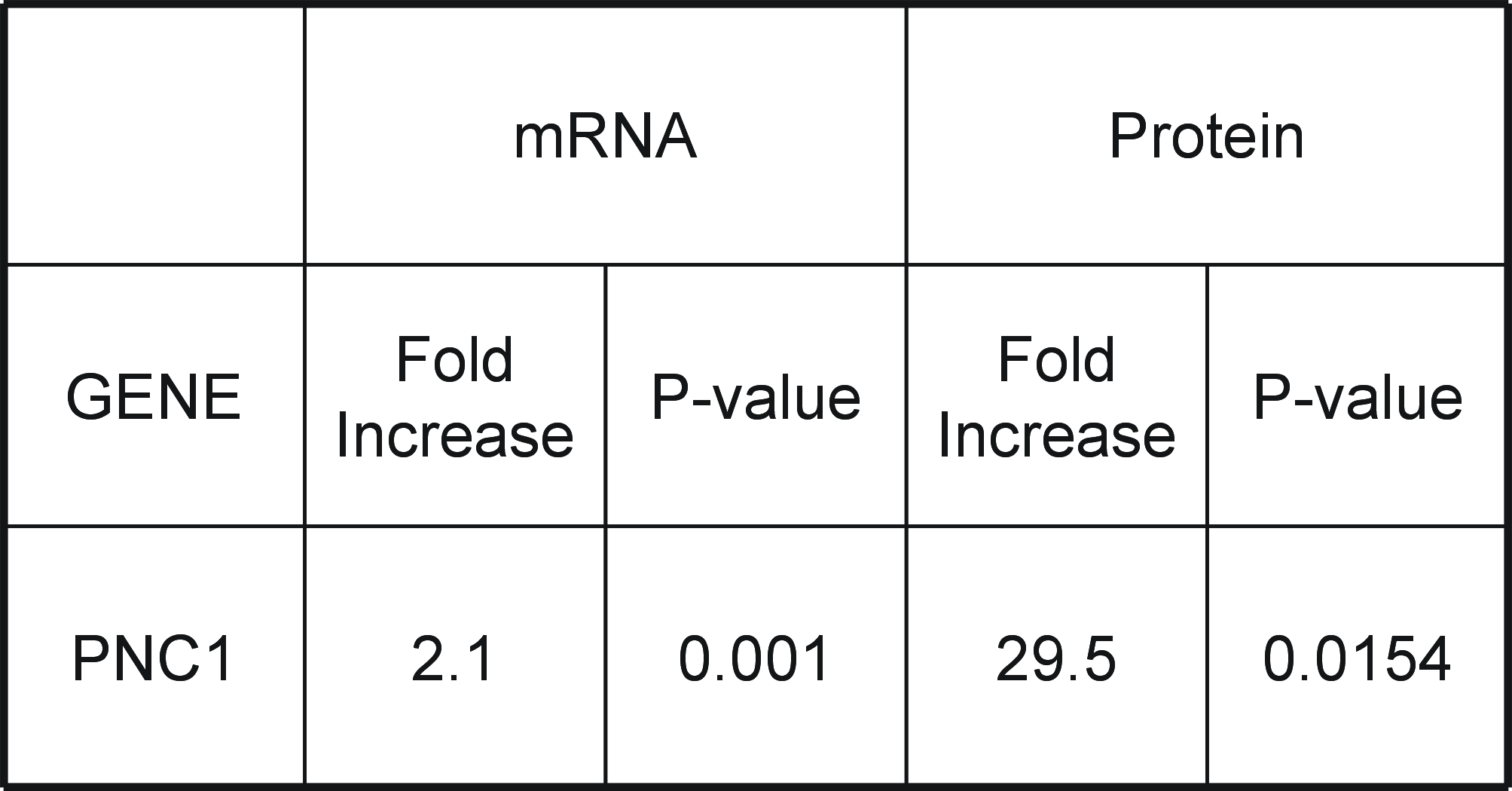

Supplement: Figure S1 — Comparison between transcriptomics and proteomics data on PNC1 expression in CUG mistranslating cells. DNA-microarray analysis showed that the PNC1 gene is induced 2-fold (data deposited in ArrayExpress http://www.ebi.ac.uk/arrayexpress/), whereas 2D-PAGE detected 30-fold increase in Pnc1p expression, suggesting that there is translational control of gene expression in CUG mistranslating cells. Microarray analyses were performed with 6 independent cultures for each strain and hybridized against the reference in dye-swap (three control strain cultures labelled Cy5 and three labelled Cy3), in a total of 6 microarrays for each mutant strain, as described previously (Silva et al, 2007). Data analysis was performed using GeneSpring (Silicon Genetics) and SAM (Significance Analysis for Microarrays). Comparison of GeneSpring data (P<0.05 by Student's t-test, fold change>1.6) and SAM analysis (D = 2.15; false discovery rate = 0.001) resulted in a common set of 170 significant genes, from which 81 were selected based on the average fold change. Proteome analyses were carried out for 3 independent biological replicates and the mean spot volumes were calculated after normalization to the total spot volume of the gel. Protein P-value was calculated using Student's t-test. (8.43 MB TIF) [file pone.0005212.s001.tif]

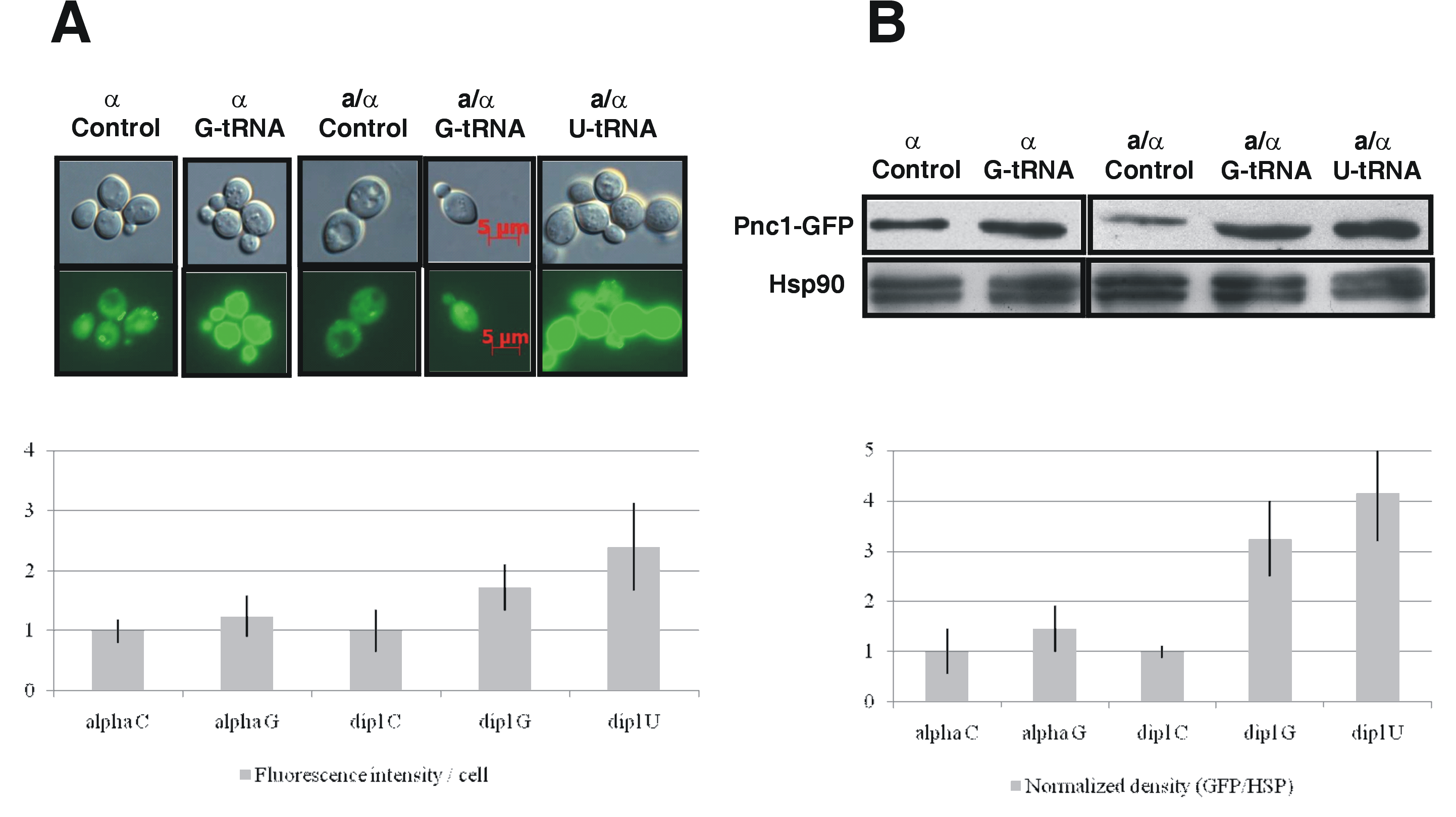

Supplement: Figure S2 — Quantification of the PNC-GFP fusion protein by densitometry. A) Fluorescence intensity was determined in the microscopy images using the AxioVision software from Zeiss. The area of each cell was delimited and the pixel intensity calculated. Values were normalized to the control and represent mean density±standard deviation. B) Western blot quantification was performed in the QuantityOne software from BioRad. Bands were delimited by boxes with similar area, and the densities calculated. After background subtraction, values of the GFP bands were normalized to the values of the HSP bands. Results are expressed as mean density±standard deviation. (1.46 MB TIF) [file pone.0005212.s002.tif]
